# Metabolically healthy across body mass index categories in relation to serum Klotho levels: a population-based study

**DOI:** 10.3389/fendo.2025.1539983

**Published:** 2025-02-21

**Authors:** Yanling Shu, Junfan Yang, Linfei Dou, Mingyang Wu

**Affiliations:** ^1^ Ministry of Education Key Laboratory of Environment and Health, and State Key Laboratory of Environmental Health (Incubating), School of Public Health, Tongji Medical College, Huazhong University of Science and Technology, Wuhan, China; ^2^ Department of Maternal and Child Health, School of Public Health, Tongji Medical College, Huazhong University of Science and Technology, Wuhan, China; ^3^ Department of Maternal and Child Health, Xiangya School of Public Health, Central South University, Changsha, China; ^4^ Xiangya School of Medicine, Central South University, Changsha, Hunan, China

**Keywords:** NHANES, Klotho, metabolically healthy, phenotypes, obesity

## Abstract

**Background:**

Metabolic health status and body mass index (BMI) are both key predictors of aging-related diseases and premature mortality. However, the relationship between metabolically distinct phenotypes, classified by BMI categories, and serum Klotho levels—a biomarker of aging—remains poorly understood. This study aimed to investigate the association between metabolically healthy phenotypes and serum Klotho levels among middle and aged adults.

**Methods:**

A total of 11,413 participants were included in this study. Participants were categorized into phenotypes according to metabolic disorders and BMI: metabolically healthy and normal weight (MH-NW), metabolically healthy and overweight (MH-OW), metabolically healthy and obesity (MHO), metabolically unhealthy and normal weight (MU-NW), metabolically unhealthy and overweight (MU-OW), and metabolically unhealthy and obesity (MUO). Weighted multivariate linear regression models were performed to estimate the association between metabolically healthy phenotypes and Klotho levels.

**Results:**

After adjusting for potential confounders, participants in the MH-OW, MU-OW, and MUO groups had significantly lower Klotho levels compared to the MH-NW group, with estimated percentage changes (95%CIs) at -5.4% (-9.2% to -1.4%), -5.0% (-8.3% to -1.5%), and -5.7% (-8.7% to -2.5%), respectively. Additionally, reduced Klotho levels were more pronounced among females with MU-OW and MUO phenotypes, showing estimated percentage changes of -6.6% (-11.1% to -1.9) and -8.4% (-12.7% to -3.8%), respectively.

**Conclusion:**

This large population-based study found that Klotho levels vary according to metabolically healthy status across BMI categories, with metabolically unhealthy phenotypes exhibiting notably lower levels. These findings highlight the influence of metabolic abnormalities and body fatness on the aging process.

## Introduction

In 1997, Kuro-o et al. (1) discovered that a defect in Klotho gene expression in mice results in a range of health conditions, including shortened life span, infertility, skin atrophy, osteoporosis and emphysema. Subsequently, in 2005, Kuro-o et al. found that overexpression of Klotho extends lifespan and acts as an aging suppressor gene in mammals (2). Klotho is generally categorized into three subfamilies: α-Klotho, β-Klotho, and γ-Klotho (3), with the term “Klotho” typically referring to α-Klotho. In humans, serum Klotho levels are higher in children and decrease with aging (4). While the precise role of Klotho remains debated, it is known to act as a cofactor for fibroblast growth factor-23, playing a key role in regulating phosphate balance and vitamin D metabolism (5). Several studies have suggested a link between Klotho and the secretion of growth hormone (GH) (6). Klotho is also known to suppress the signaling activity of insulin-like growth factor-1 (IGF-1), a known inhibitor of GH secretion, likely by disrupting receptor-ligand interactions (7). As a result, lower levels of Klotho enhance IGF-1 signaling, leading to a reduction in GH secretion. These reduced Klotho levels have been linked to decreased lifespan and various adverse health impacts in both animals and humans (8, 9). Therefore, understanding the factors that influence Klotho levels is critical for promoting healthy aging.

Previous studies have investigated the relationship between obesity and IGF-1 levels and found a dysregulation of IGF-1 pathways in individuals with obesity (10), suggesting a potential link between body fat and Klotho. Recent evidence has shown Klotho to be lower in adults with obesity compared to their normal-weight counterparts (11). A large-scale population-based study further indicated that obesity is associated with decreased Klotho levels, with significant differences in Klotho concentrations observed in adults who underwent weight loss interventions, correlating with the extent of weight loss achieved (12, 13). Although obesity is a well-established risk factor for various health conditions, the “obesity paradox” suggests that not all individuals with obesity exhibit the typical metabolic abnormalities (14). Som individuals with overweight or obesity seem to have a better metabolic profile and physical health, a condition referred to as “metabolically healthy obesity” (MHO) (15). Specifically, the MHO or metabolically healthy overweight (MH-OW) phenotype is characterized by high BMI and insulin sensitivity without elevated blood pressure or serum lipids (16–18). Some studies have linked MHO or MH-OW to increased cardiovascular disease risks (19, 20), while others argue that these phenotypes can be considered relatively healthy (21–23). The primary mechanism connecting adiposity traits to accelerated aging is thought to involve increased oxidative stress and low-grade inflammation (24). Klotho has been proposed as a regulator of oxidative stress and cellular senescence (25), and its expression is also modulated by oxidative stress and low-grade inflammation (26). Given the strong associations of adiposity traits with oxidative stress, low-grade inflammatory and aging-related diseases, it is reasonable to hypothesize that metabolic abnormalities across different body fat levels might have varying effects on the aging process. Moreover, due to Klotho’s potential involvement in aging, exploring its relationship with different metabolically healthy phenotypes is crucial for advancing our understanding of healthy aging. However, no research to date has specifically examined the connection between metabolically healthy phenotypes and Klotho.

Therefore, our study aims to address this gap by investigating the relationship between metabolically healthy across BMI categories and serum Klotho levels. To achieve this, we utilized data from the National Health and Nutrition Examination Survey (NHANES), which provides comprehensive information on serum Klotho levels (referred to as Klotho in this study) measured during the survey (27, 28).

## Methods

### Data sources

The National Health and Nutrition Examination Survey (NHANES) is a cross-sectional study designed to assess the health and nutrition status of adults and children in the United States (27–30). The survey combines interviews and physical exams. NHANES is a major program of the National Center for Health Statistics (NCHS). The NCHS is part of the Centers for Disease Control and Prevention (CDC) and is responsible for providing the nation with vital health statistics. In 1999, the survey became a continuing program with a shift in focus to various health and nutrition measures to meet emerging needs. Because a multi-stage stratified probability sampling method was used in NHANES, approximately 5,000 people are selected as a nationally representative sample to be surveyed and examined. The survey is conducted every two years as a repeat cycle for data collection. The protocol was approved by the NCHS Institutional Review Board, and each participant provided a written informed consent. Source of data and use: [NHANES - National Health and Nutrition Examination Survey Homepage (cdc.gov)].

### Study population

For the present study, data on 50,588 participants were obtained from the NHANES database during five consecutive cycles from 2007 to 2016. First, participants without serum Klotho data (n= 36,824) were excluded. Next, participants without BMI information or metabolic health information were excluded (n= 168). Then, individuals with incomplete covariate information (including age, sex, race/ethnicity, education level, poverty-to-income ratio (PIR), smoking status, and alcohol consumption) were excluded from this analysis (N=2183). Finally, a total of 11,413 participants were included in the current analysis.

### Assessment of metabolically healthy phenotypes

Weight status is categorized according to the BMI, which is calculated by dividing weight (in kilograms) by the square of height (in meters). In this study, BMI criteria are divided into normal weight (BMI18.5 to 24.9 kg/m^2^), overweight (BMI25.0 to 29.9 kg/m^2^) and obesity (BMI≥30 kg/m^2^) according to the criteria of the US standards (31). Individuals containing at least one of four metabolic indicators were categorized as metabolically unhealthy, including: (i) hypertension (self-reported diagnosed hypertension, taking prescription for hypertension, SBP≥130 or DBP≥85); (ii)diabetes mellitus (self-reported diagnosed diabetes, taking prescription to lower blood sugar or taking insulin, HbA1c≥5.7%, fasting plasma glucose≥100mg/dL, or OGTT-2h≥140mg/dL); (iii) abnormal HDL (male: HDL cholesterol <40 mg/dL; Women <50 mg/dL); (iv)LDL >150 mg/dL or use of lipid-lowering drugs. Therefore, six metabolically healthy phenotypes according to weight status were generated, including metabolically healthy normal weight (MH-NW), metabolically healthy overweight (MH-OW), metabolically healthy obesity (MHO), metabolically unhealthy normal weight (MU-NW), metabolically unhealthy overweight (MU-OW), and metabolically unhealthy obesity (MUO) (32).

### Determination of serum klotho levels

In this study, frozen serum samples from individuals aged 40 to 79 years were collected during five cycles at NHANES from 2007 to 2016 to measure serum Klotho concentrations. Serum Klotho concentration analysis was performed using a commercial ELISA kit (IBL International, Japan) (3, 4, 27). All samples were stored at −80°C prior to examination. Subsequently, the samples were shipped from the Centers for Disease Control and Prevention to the Northwestern Lipid Metabolism and Diabetes Research Laboratory at the University of Washington for further analysis. Quality assurance measures included averaging the results from two repeated analyses. Samples with more than a 10% difference between the repeated results were reanalyzed, and quality control samples with more than two standard deviations are considered invalid for the entire test plate. The detection sensitivity of serum Klotho concentration was 4.33 pg/mL. To establish a reference range, a set of 114 samples from healthy donors was evaluated, resulting in a range of 285.8 to 1638.6 pg/mL, with an average concentration of 698.0 pg/mL (3, 33). For more detailed Klotho instructions for testing, please refer to the NHANES website: NHANES - National Health and Nutrition Examination Survey Homepage (cdc.gov).

### Covariates and data collection

Based on previous studies (34–39), relevant covariates included sex, race/ethnicity, educational attainment, age, PIR, serum cotinine, alcohol drinking, and estimated glomerular filtration rates (eGFR). A standardized questionnaire was used to obtain the baseline characteristics of each participant, including age, sex, race/ethnicity, educational attainment, alcohol drinking, and history of diabetes and hypertension. Serum cotinine levels, used to estimate exposure to environmental tobacco smoke, were measured by an isotope dilution-high performance liquid chromatography/atmospheric pressure chemical ionization tandem mass spectrometry. We used the serum creatinine level to estimate the eGFR according to the CKD Epidemiology Collaboration Equations (40).

### Statistical analysis

Categorical variables are presented in frequency and percentage terms, and continuous variables with normal or skew distributions are described by mean ± standard deviation or median (IQR). The Klotho levels were log-transformed to an approximate normal distribution due to its highly skewed distribution. Participants’ baseline characteristics are listed according to metabolically healthy phenotypes. For baseline characteristics analysis, Chi-square tests were used for categorical variables, ANOVA for continuous variables with normal distribution, and non-parametric tests for continuous variables with skewed distributions.

To estimate the association between metabolically healthy phenotypes and Klotho levels, weighted multivariate linear regression models were carried out using R software (svyglm function). Due to the log-transformed Klotho levels in models, in order to make the results more intuitive, we calculated the percentage change in serum Klotho concentrations for different categories of metabolically healthy phenotypes according to the following formula: (e^(β±1.96×SE)^ − 1) × 100%, where β and SE represent the regression coefficient and standard error, respectively. To minimum the bias by potential covariates, three models were used in this study. Model 1 was a crude model with no adjustment for any covariate. Model 2 was a minimally adjusted model, with covariates sex and age included for adjustment. Model 3 was a fully adjustment model, with covariates age, sex, ethnicity/race, educational attainment, PIR, eGFR, cotinine and alcohol consumption included for analysis.

In the sensitivity analysis, three models were additionally conducted. First, missing covariate data were imputed to determine whether the complete case analyses were biased. Multiple imputations by chained equations with 5 imputations were used to impute missing values (“mice” package in R). Second, to better account for the potential impact of fat distribution, we replaced BMI-based obesity with waist circumference-based abdominal obesity. Abdominal obesity was then combined with metabolic health status to form different phenotypes, and its association with Klotho levels was analyzed. Finally, to account for the potential influence of dietary habits, physical activity, and comorbidities on Klotho levels, we further incorporated these variables into the models.

Based on the close correlation between age and Klotho concentration shown in previous studies (4, 11, 41) and the potential sex differences in aging process. Therefore, this study conducted subgroup analyses to further explore the potential effects of different subgroups on the relationship between metabolically healthy phenotypes and serum Klotho levels. R software (version 4.2.2.) was used for all data analysis, and p<0.05 was considered statistically significant.

## Result

**Table 1** presents the baseline characteristics of all participants in terms of metabolically healthy phenotypes. A total of 11,413 participants (males, 49.2%) were included in the study. There were significant differences in demographic characteristics, lifestyles, and health conditions across metabolically healthy phenotypes (p<0.05). Among metabolically healthy versus metabolically unhealthy individuals, normal-weight individuals (MH-NW and MU-NW) tended to have higher Klotho levels, while individuals with obesity (MHO and MUO) had lower serum Klotho levels.

**Table 1:** Baseline characteristics of different metabolically healthy phenotypes across BMI categories among study participants (n=11413).

Characteristics	MH-NW	MH-OW	MHO	MU-NW	MU-OW	MUO	*P*
Age, years, mean (sd)	52.2 (9.7)	52.1 (9.5)	52.3 (9.9)	59.5 (10.8)	59.3 (10.8)	58 (10.5)	<0.001
BMI, kg/m^2^, mean (sd)	22.1 (2)	27.3 (1.4)	34.4 (4.5)	22.6 (1.9)	27.5 (1.4)	35.9 (5.6)	<0.001
Sex, n(%)							
men	321 (42.1)	372 (54.5)	184 (42.2)	889 (46.7)	1895 (57.5)	1953 (45.1)	<0.001
women	441 (57.9)	310 (45.5)	252 (57.8)	1013 (53.3)	1401 (42.5)	2382 (54.9)	<0.001
Educational attainment, n(%)							
<High school	143 (18.8)	135 (19.8)	116 (26.6)	502 (26.4)	938 (28.5)	1188 (27.4)	<0.001
High school	125 (16.4)	138 (20.2)	76 (17.4)	421 (22.1)	755 (22.9)	1029 (23.7)	<0.001
College or above	494 (64.8)	409 (60)	244 (56)	979 (51.5)	1603 (48.6)	2118 (48.9)	<0.001
Race/Ethnicity, n(%)							
Non Hispanic White	421 (55.2)	333 (48.8)	188 (43.1)	843 (44.3)	1466 (44.5)	1922 (44.3)	<0.001
Non Hispanic Black	93 (12.2)	99 (14.5)	84 (19.3)	371 (19.5)	584 (17.7)	1018 (23.5)	<0.001
Other Hispanic	55 (7.2)	68 (10)	55 (12.6)	183 (9.6)	397 (12)	476 (11)	<0.001
Mexican American or Other	193 (25.3)	182 (26.7)	109 (25)	505 (26.6)	849 (25.8)	919 (21.2)	<0.001
Serum cotinine, median(IQR)	0 (0-85.2)	0 (0-0.2)	0 (0-0.3)	0 (0-179.5)	0 (0-2.6)	0 (0-0.7)	<0.001
Alcohol drinking, n(%)							
more than 12 drinks/yr	181 (23.8)	144 (21.1)	118 (27.1)	546 (28.7)	885 (26.9)	1392 (32.1)	<0.001
less than 12 drinks/yr	581 (76.2)	538 (78.9)	318 (72.9)	1356 (71.3)	2411 (73.1)	2943 (67.9)	<0.001
Metabolically unhealthy, Diabetes, n(%)							
no	762 (100)	682 (100)	436 (100)	1356 (71.3)	2468 (74.9)	3321 (76.6)	<0.001
yes	0 (0)	0 (0)	0 (0)	546 (28.7)	828 (25.1)	1014 (23.4)	<0.001
Metabolically unhealthy, Hypertension, n(%)							
no	762 (100)	682 (100)	436 (100)	610 (32.1)	948 (28.8)	882 (20.3)	<0.001
yes	0 (0)	0 (0)	0 (0)	1292 (67.9)	2348 (71.2)	3453 (79.7)	<0.001
Metabolically unhealthy, HDL, n(%)							
no	762 (100)	682 (100)	436 (100)	1470 (77.3)	2127 (64.5)	2306 (53.2)	<0.001
yes	0 (0)	0 (0)	0 (0)	432 (22.7)	1169 (35.5)	2029 (46.8)	<0.001
Metabolically unhealthy, LDL, n(%)							
no	762 (100)	682 (100)	436 (100)	1216 (63.9)	1913 (58)	2487 (57.4)	<0.001
yes	0 (0)	0 (0)	0 (0)	686 (36.1)	1383 (42)	1848 (42.6)	<0.001
eGFR, mean (sd)	97.2 (23.2)	97 (23.1)	101.6 (25.4)	91.5 (27.4)	89.4 (26)	89.8 (27.5)	<0.001
Klotho, pg/mL, median (IQR)	863 (699.1-1054.3)	802.5 (672.2-1000)	829 (669.8-993.2)	805.1 (657.2-989.7)	791.3 (648-974.6)	796.8 (646-988)	<0.001

BMI, body mass index; eGFR, estimated glomerular filtration rate; IQR, interquartile range; HDL, high-density lipoprotein; LDL, low-density lipoprotein.; MH-NW, metabolically healthy normal weight; MH-OW, metabolically healthy overweight; MHO, metabolically healthy obesity; MU-NW, metabolically unhealthy normal weight; MU-OW, metabolically unhealthy overweight; MUO, metabolically unhealthy obesity.

**Table 2** presents the association between Klotho levels and metabolically healthy phenotypes. In the unadjusted model, as compared with the MH-NW group, participants in the MH-OW, MU-NW, MU-OW and MUO groups had significantly lower Klotho levels, with a percentage change (95%CI) of -5.6% (-9.5% to -1.5%), -4.4% (-7.6% to -1.1%), -6.7% (-10.0% to -3.3%) and -6.5% (-9.6% to –3.3%), respectively. After adjusting for age, gender, educational attainment, eGFR, race/ethnicity, serum levels of cotinine, and alcohol drinking, compared with MH-NW group, participants in the MH-OW, MU-OW, and MUO had significant decreased Klotho levels, with estimated percentage changes (95%CIs) at -5.4% (-9.2% to -1.4%), -5.0% (-8.3% to -1.5%), and -5.7% (-8.7% to -2.5%), respectively. Moreover, to avoid potential bias by missing value on covariates, multiple imputations by chained equations with 5 imputations were used to impute missing values. In the imputed dataset, the association between Klotho levels and metabolically healthy phenotypes was not changed materially (-6.2 [-9.0 to -3.3], **Supplementary Table S1**). To better account for the potential impact of fat distribution, we replaced BMI-based obesity with waist circumference-based abdominal obesity, similar findings were observed in the effects of metabolically healthy phenotypes on serum Klotho levels (MUO: -4.4% [-7.3% to -1.4%], **Supplementary Table S2**). After further adjusted for dietary energy intake, physical activity, and cardiovascular diseases, the association between metabolically healthy phenotypes was not changed materially (-6.1 [-9.1 to -3.0], **Supplementary Table S3**).

**Table 2:** The association between serum Klotho levels and metabolically healthy phenotypes across BMI categories.

Metabolically healthy phenotypes	Model 0		Model 1		Model 2	
	Percent Changes (%) and 95%CI	*P*	Percent Changes (%) and 95%CI	*P*	Percent Changes (%) and 95%CI	*P*
MH-NW	Ref		Ref		Ref	
MH-OW	-5.6 (-9.5, -1.5)	0.008	-5.1 (-9.0, -1.0)	0.015	-5.4 (-9.2, -1.4)	0.009
MHO	-3.5 (-8.7, 2.1)	0.210	-3.0 (-8.3, 2.6)	0.282	-4.4 (-9.6, 1.2)	0.121
MU-NW	-4.4 (-7.6, -1.1)	0.011	-2.7 (-6.0, 0.6)	0.106	-2.8 (-6.0, 0.5)	0.091
MU-OW	-6.7 (-10.0, -3.3)	<0.001	-4.7 (-8.1, -1.1)	0.011	-5.0 (-8.3, -1.5)	0.006
MUO	-6.5 (-9.6, -3.3)	<0.001	-5.0 (-8.1, -1.7)	0.003	-5.7 (-8.7, -2.5)	<0.001

MH-NW, metabolically healthy normal weight; MH-OW, metabolically healthy overweight; MHO, metabolically healthy obesity; MU-NW, metabolically unhealthy normal weight; MU-OW, metabolically unhealthy overweight; MUO, metabolically unhealthy obesity.

Model 0: crude model.

Model 1: adjusted for age and sex.

Model 2: adjusted for age, sex, education, eGFR, race/ethnicity, serum cotinine and alcohol drinking.

**Table 3** presents the associations of metabolically healthy phenotypes with Klotho levels stratified by sex and age. After adjusting for potential confounders, the decreased Klotho levels were more prominent in participants with phenotypes of MHO, MU-OW and MUO in the females, with estimated percentage changes of -7.9% (-14.5 to -0.8), -6.6% (-11.1 to -1.9) and -8.4% (-12.7%to -3.8%), respectively. In addition, the decreased Klotho levels were more prominent in participants with phenotypes of MH-OW, MU-NW, MU-OW and MUO in older adults (≥60 years), with estimated percentage change (95%CI) of -10.1% (-18.8% to -0.4%), -7.5% (-12.5% to -2.2%), -10.4% (-15.5% to -5.0%), and -9.9% (-14.8% to -4.8%), respectively. In participants with age <60 years, the decreased Klotho levels were more significant in participants with MUO phenotype, and the estimated percentage change (95%CI) is -4.7% (-8.2% to -1.1%).

**Table 3:** The associations between metabolically healthy phenotype across BMI categories and Klotho levels, stratified by sex and age.

Metabolically healthy phenotypes	Sex ^a^		Age ^b^	
	Percent Changes (%) and 95% CI	*P*	Percent Changes (%)and 95% CI	*P*
	males		age<60	
MH-NW	Ref		Ref	
MH-OW	-4.5 (-9.8, 1.2)	0.120	-4.5 (-8.8, -0.1)	0.046
MHO	0.2 (-6.8, 7.8)	0.950	-4.9 (-10.4, 0.8)	0.089
MU-NW	-1.1 (-5.5, 3.5)	0.623	-1.7 (-5.8, 2.5)	0.411
MU-OW	-2.3 (-6.8, 2.4)	0.327	-3.3 (-7.5, 1.1)	0.133
MUO	-1.9 (-6.3, 2.7)	0.410	-4.7 (-8.2, -1.1)	0.012
	females		Age≥60	
MH-NW	Ref		Ref	
MH-OW	-5.3 (-11.8, 1.8)	0.136	-10.1 (-18.8, -0.4)	0.043
MHO	-7.9 (-14.5, -0.8)	0.029	-3.8 (-12.7, 5.9)	0.423
MU-NW	-3.6 (-8.2, 1.2)	0.139	-7.5 (-12.5, -2.2)	0.007
MU-OW	-6.6 (-11.1, -1.9)	0.007	-10.4 (-15.5, -5.0)	<0.001
MUO	-8.4 (-12.7, -3.8)	<0.001	-9.9 (-14.8, -4.8)	<0.001

MH-NW, metabolically healthy normal weight; MH-OW, metabolically healthy overweight; MHO, metabolically healthy obesity; MU-NW, metabolically unhealthy normal weight; MU-OW, metabolically unhealthy overweight; MUO, metabolically unhealthy obesity.

a adjusted for age, ethnicity, PIR, eGFR, educational attainment, serum cotinine, alcohol drinking.

b adjusted for sex, ethnicity, PIR, eGFR, educational attainment, serum cotinine, alcohol drinking.

## Discussion

Using large population-based data, we found that serum Klotho levels varied according to metabolically healthy phenotypes. In the fully adjusted model, Klotho levels were significantly lower in the MH-OW, MU-OW, and MUO groups compared with the MH-NW group. Notably, the association between the MU phenotypes and decreased Klotho levels was more significant in people over 60 years of age, while these associations (except MUO) was not significantly different in people under 60 years of age. In addition, the association between MU-OW and MUO phenotypes and decreased Klotho levels was more pronounced in female participants.

Previous studies have shown that metabolically healthy phenotypes were associated with many long-term health problems (19, 20, 42, 43). In the existing studies on metabolically healthy phenotypes and cardiovascular events, persistent MHO and MU-OW have a higher risk of cardiovascular disease (CVD) compared with MH-NW group (19, 20). Furthermore, unstable MHO transitioning from metabolically healthy to MUO also increases the risk of CVD (42). In addition, compared with MH-NW, MHO and MH-OW individuals had an increased risk of cancer (38, 44). In one previous study focused on chronic kidney disease (CKD), MUO had the highest risk of CKD, followed by MHO (43). These studies suggested that metabolically healthy across BMI categories may result in different health risks, although the mechanism is unclear. Our study is the first population-based study on the relationship between different metabolically healthy phenotypes and Klotho. Results from this study demonstrated that Klotho levels of MH-OW, MU-OW, and MUO metabolically healthy phenotypes are significantly decreased compared with MH-NW. These findings suggest that different metabolically healthy phenotypes may pose a threat to health by reducing the level of Klotho. Although this is the first to find an association between metabolically healthy phenotype and Klotho among a nationwide population and the findings of this study may help explain the relationship between metabolic disorders and long-term diseases or premature mortality, it is important to note that evidence in humans remains limited. This highlights the need for further research to fully clarify the mechanistic role of Klotho and its clinical significance.

The potential mechanisms underlying the association between metabolically healthy phenotypes and Klotho are not yet fully understood, but they may be related to chronic inflammation caused by obesity or metabolic disorders. In general, obesity is often accompanied by increased levels of inflammation in the body, which can damage cells and tissues, and affect the synthesis and secretion of Klotho (33, 45). The Klotho protein has anti-inflammatory effects (46), and a decrease in its levels may exacerbate inflammatory response, creating a vicious cycle. Moreover, obesity and poor metabolically healthy are often linked to conditions such as such as insulin resistance and diabetes, which may impact Klotho levels by altering the endocrine environment and metabolic pathways. For example, insulin resistance and diabetes can disrupt the normal function of Klotho, leading to reduced expression in the body (8, 47). Additionally, obesity can lead to increased visceral fat, which secretes adipokines (such as adiponectin) that may negatively affect Klotho level (48). Abnormal secretion of these adipokines can influence the synthesis and function of the Klotho protein, further reducing its levels (17, 49). Therefore, obesity and metabolic disorders affect the relationship between metabolically healthy phenotypes and Klotho through mechanisms such as inducing chronic inflammation, interfering with insulin function and abnormal secretion of adipokines. Given the current lack of comprehensive studies on these mechanisms, further experimental research is needed to validate the findings from our study.

Interestingly, our study reveals that among elderly individuals with metabolically unhealthy profiles, Klotho levels were significantly decreased even when they maintained a normal weight. Conversely, in the middle-aged population, it was only the MUO individuals who exhibited a notable decline in Klotho levels. Although the specific mechanism is unclear, the observed differences might highlight the importance of normal weight among middle aged adults. We speculate that the following reasons might be responsible for these findings: Generally, various physiological functions (such as hormone secretion and metabolic rate) of the human body gradually decline with age (50). But middle-aged individuals being relatively younger, possess more robust metabolic functions. It is typically only when severe metabolic abnormalities occur, such as being metabolically unhealthy and obesity (e.g., MUO), that a significant decrease in Klotho levels is observed. However, in the elderly, due to the already declined metabolic function, even a normal weight may not protect against the adverse effects of an unhealthy metabolic state on accelerated aging. This unhealthy metabolic state can further exacerbate the decline in Klotho levels, creating a cumulative effect that results in notably lower Klotho levels compared to their metabolically healthy counterparts of the same age. Due to the complex interactions between age, Klotho and metabolically healthy, future research may need to further explore the mechanisms underlying Klotho’s role in metabolically healthy across different age groups to provide a deeper understanding. Additionally, in this study, we found a significant negative correlation between metabolically unhealthy phenotypes and Klotho levels, with this association being particularly pronounced in women. Several factors may contribute to this gender difference. First, women and men have distinct fat distribution patterns, with women typically storing more subcutaneous fat in the lower body, while an increase in visceral fat is closely linked to a decline in Klotho levels (9, 48). Second, estrogen plays a key role in regulating fat distribution and metabolic health in women. A decrease in estrogen levels, especially after menopause, may lead to fat accumulation and metabolic dysregulation, which in turn reduces Klotho expression (51). Furthermore, women with metabolically unhealthy phenotypes are likely to experience more severe chronic low-grade inflammation, which is also associated with lower Klotho levels. Additionally, since the participants in this study were middle-aged and older adults, many of the women were either peri-menopausal or postmenopausal, where hormonal changes may accelerate biological aging, contributing to the significant decline in Klotho levels observed in these women. Taken together, the interplay of factors such as fat distribution, hormonal fluctuations, and inflammation may explain the more pronounced association between metabolically unhealthy phenotypes and declined Klotho levels in women. These results imply that the influence of demographic factors should be considered in public health strategies. With the improvement of living standards, people are paying more and more attention to longevity. This reminds us that a higher BMI may have a greater effect on longevity in metabolically unhealthy conditions, even in metabolically healthy conditions. Future studies on the influence of metabolically healthy phenotypes on Klotho levels and considering the role of Klotho as a longevity factor would provide new insights for healthy aging and early risk assessment.

Notably, the present findings appear to challenge the concept of the “obesity paradox”. The “obesity paradox” refers to the phenomenon where individuals with higher BMI in certain populations seem to experience better survival rates and health outcomes compared to those with lower BMI, despite the well-established risks of obesity in the general population (14). This paradox is particularly relevant in contexts such as cardiovascular disease, diabetes, and aging (14). Recent studies have suggested that obesity, when combined with metabolic health (i.e., absence of insulin resistance, dyslipidemia, and hypertension), may not be as detrimental as previously thought and may even confer certain protective effects, especially in older adults (52). However, in the present study, although the association between MHO and Klotho levels did not reach statistical significance, the point estimate was still negative, suggesting that MHO may have a negative effect on Klotho levels, rather than offering a protective effect. Recent studies have indicated that higher Klotho levels may provide greater protection against mortality (53, 54). Given these findings, the observed decrease in Klotho levels among individuals with MHO in this study could indicate that this condition might also accelerate the aging process, thereby failing to provide protection against mortality risk. Therefore, maintaining a metabolically healthy and normal weight status may still be an important strategy for middle-aged and older adults to combat accelerated aging.

Our study has several limitations. First, since this study uses a cross-sectional design, we can only observe the correlation between Klotho levels and metabolically healthy phenotypes, without being able to infer causality. While we hypothesize that metabolically unhealthy obesity may affect Klotho levels through low-grade inflammation or oxidative stress, the cross-sectional design still leaves room for reverse causality. Therefore, it is not possible to definitively determine whether low Klotho levels cause metabolic dysfunction, or whether metabolic disturbances lead to a decrease in Klotho levels. Future longitudinal or experimental studies could further investigate the interrelationship between Klotho and metabolically healthy phenotypes, helping to uncover the underlying causal mechanisms. Second, our study population consists of individuals aged 40-79 years, so the findings may not be generalizable to younger age groups. Third, our definitions of overweight and obesity were based solely on BMI; including body fatness or other body shape indexes-based definitions could provide additional insights. Finally, while we accounted for various covariates, some potential unmeasured confounders may still challenge the present findings.

Revealing the associations of metabolically healthy phenotypes with Klotho levels has significant public health or clinical implications. Against the backdrop of an aging population, life expectancy is steadily increasing across countries, accompanied by a growing burden of age-related diseases. The results of this study suggest that maintaining metabolic health and normal body weight remains an effective strategy in combating aging or aging-related diseases. At the individual level, for those already exhibiting a metabolically unhealthy obese phenotype, it remains unclear whether increasing Klotho protein levels could further delay aging and reduce the long-term risk of chronic diseases. This question warrants further exploration in future longitudinal studies and clinical trials.

## Conclusion

In this large population-based study, Klotho levels were varied according to metabolically healthy status across BMI categories, with metabolically unhealthy phenotypes showed more decreased levels. These findings highlight the impact of metabolic abnormalities across body fatness on the aging process.

## Data Availability

The raw data supporting the conclusions of this article will be made available by the authors, without undue reservation.
