# Supplementary material for: Metabolically healthy across body mass index categories in relation to serum Klotho levels: a population-based study
Source: Front Endocrinol (Lausanne). 2025 Feb 21;16:1539983. doi: 10.3389/fendo.2025.1539983 (PMC11885134; doi:10.3389/fendo.2025.1539983)
Supplement: Supplementary file 1 [file Table1.docx]

**Metabolically healthy across body mass index categories in relation to serum Klotho levels: A population-based study**

Yanling Shu,^1,2,✝^, Junfan Yang,^3,4,✝^, Linfei Dou,^3^ Mingyang Wu,^3,*^

**Table S1** The association between metabolically healthy phenotypes and serum Klotho levels (imputation dataset).

| Metabolically healthy phenotypes | Model 0 | | Model 1 | | Model 2 | |
| --- | --- | --- | --- | --- | --- | --- |
|  | Percent Changes (%) and 95%CI | *P* | Percent Changes (%) and 95%CI | *P* | Percent Changes (%) and 95%CI | *P* |
| MH-NW | Ref | Ref | Ref | Ref | Ref | Ref |
| MH-OW | -6.0 (-10.1, -1.7) | 0.008 | -5.3 (-9.4, -1.0) | 0.017 | -5.6 (-9.8, -1.3) | 0.012 |
| MHO | -6.1 (-10.1, -2.0) | 0.005 | -5.6 (-9.6, -1.4) | 0.010 | -6.6 (-10.5, -2.5) | 0.002 |
| MU-NW | -5.8 (-8.8, -2.8) | <0.001 | -4.0 (-7.1, -0.8) | 0.014 | -3.9 (-6.9, -0.7) | 0.016 |
| MU-OW | **-8.1 (-10.9, -5.1)** | **<0.001** | **-5.9 (-8.9, -2.8)** | **<0.001** | **-6.0 (-8.9, -2.9)** | **<0.001** |
| MUO | **-7.5 (-10.3, -4.6)** | **<0.001** | **-5.8 (-8.6, -2.8)** | **<0.001** | **-6.2 (-9.0, -3.3)** | **<0.001** |

**Note:** MH-NW, metabolically healthy normal weight; MH-OW, metabolically healthy overweight; MHO, metabolically healthy obesity; MU-NW, metabolically unhealthy normal weight; MU-OW, metabolically unhealthy overweight; MUO, metabolically unhealthy obesity.

Model 0: crude model.

Model 1: adjusted for age and sex.

Model 2: adjusted for age, sex, education, eGFR, race/ethnicity, serum cotinine and alcohol drinking.

**Table S2** The association between metabolically healthy phenotypes and serum Klotho levels (waist circumference-based abdominal obesity)

| Metabolically healthy phenotypes | Model 0 | | Model 1 | | Model 2 | |
| --- | --- | --- | --- | --- | --- | --- |
|  | Percent Changes (%) and 95%CI | *P* | Percent Changes (%) and 95%CI | *P* | Percent Changes (%) and 95%CI | *P* |
| MH-NW | Ref |  | Ref |  | Ref |  |
| MHO | -3.7 (-7.5, 0.4) | 0.074 | -3.8 (-7.7, 0.2) | 0.064 | -4.0 (-7.8, -0.0) | 0.048 |
| MU-NW | -4.2 (-7.0, -1.3) | 0.006 | -2.4 (-5.3, 0.5) | 0.106 | -2.4 (-5.2, 0.6) | 0.116 |
| MUO | **-5.4 (-8.2, -2.5)** | **<0.001** | **-4.2 (-7.1, -1.2)** | **0.007** | **-4.4 (-7.3, -1.4)** | **0.004** |

**Note:** the cut-off value for waist circumference-based abdominal obesity: 102cm for men and 88cm for women.

MH-NW, metabolically healthy normal weight; MH-OW, metabolically healthy overweight; MHO, metabolically healthy obesity; MU-NW, metabolically unhealthy normal weight; MU-OW, metabolically unhealthy overweight; MUO, metabolically unhealthy obesity.

Model 0: crude model.

Model 1: adjusted for age and sex.

Model 2: adjusted for age, sex, education, eGFR, race/ethnicity, serum cotinine and alcohol drinking.

**Table S3** The association between metabolically healthy phenotypes and serum Klotho levels

| Metabolically healthy phenotypes | Model | |
| --- | --- | --- |
|  | Percent Changes (%) and 95%CI | *P* |
| MH-NW | Ref |  |
| MH-OW | **-5.3 (-9.7, -0.7)** | **0.024** |
| MHO | **-5.3 (-9.5, -0.9)** | **0.019** |
| MU-NW | **-3.7 (-6.9, -0.3)** | **0.032** |
| MU-OW | **-5.8 (-9.1, -2.4)** | **0.001** |
| MUO | **-6.1 (-9.1, -3.0)** | **<0.001** |

**Note:** MH-NW, metabolically healthy normal weight; MH-OW, metabolically healthy overweight; MHO, metabolically healthy obesity; MU-NW, metabolically unhealthy normal weight; MU-OW, metabolically unhealthy overweight; MUO, metabolically unhealthy obesity.

**Model: adjusted for age, sex, education, eGFR, race/ethnicity, serum cotinine, alcohol drinking, dietary energy intake, physical activity, and cardiovascular diseases.**
